# Association between nutritional status and health perception among participants of the Academida da Saúde Program: a cross-sectional study, Belo Horizonte, 2013-2014

**DOI:** 10.1590/S2237-96222026v35e20250992.en

**Published:** 2026-07-18

**Authors:** Tatiane Moreira Alves Garcia, Maria Cecília Ramos de Carvalho, Aline Cristine Souza Lopes

**Affiliations:** 1 Universidade Federal de Minas Gerais, Belo Horizonte, MG, Brazil Universidade Federal de Minas Gerais Belo Horizonte MG Brazil

**Keywords:** Diagnostic Self Evaluation, Obesity, Health Promotion, Primary Health Care, Cross-Sectional Studies, Autoevaluación Diagnóstica, Obesidad, Promoción de la Salud, Atención Primaria de Salud, Estudios Transversales.

## Abstract

**Objective:**

To analyze the association between nutritional status and health perception among participants of the Health Academy Program (*Programa Academia da Saúde*) in Belo Horizonte, Minas Gerais, Brazil.

**Methods:**

A cross-sectional study using baseline data from a randomized controlled community trial conducted with a representative sample of the Program. Individuals aged 20 years or older answered a questionnaire including sociodemographic variables, self-reported morbidities, and health perception, and had their nutritional status assessed. For data presentation, absolute and relative frequencies and 95% confidence intervals (95%CI) were used. Regression adjustments were performed to assess the association between nutritional status (underweight or normal weight, overweight, and obesity) and health perception (good or very good, and very poor, poor, or fair), and odds ratios (OR) with 95%CI were used as measures of association.

**Results:**

Of the 3,264 participants, 2,827 (88.0%) were women and 1,931 (56.6%) were adults; 1,352 (41.4%) were overweight, and 944 (28.9%) had obesity; 1,730 (53.0%) reported hypertension, 1,433 (44.3%) dyslipidemia, and 548 (16.8%) diabetes. Regarding health perception, 2,358 (72.3%) respondents rated their health as good or very good. Individuals with obesity were found to have 47.0% higher odds of reporting very poor, poor, or fair health perception compared to their counterparts.

**Conclusion:**

Obesity was associated with poorer health perception among participants in the Health Academy Program.

## Resumen

**Objetivo:**

Analizar la asociación entre el estado nutricional y la percepción de salud de los participantes del Programa Academia de la Salud (*Programa Academia da Saúde*) de Belo Horizonte, Minas Gerais.

**Métodos:**

Estudio transversal con datos de la línea de base de un ensayo comunitario controlado aleatorizado realizado en una muestra representativa del Programa. Individuos de 20 años o más respondieron un cuestionario que incluyó variables sociodemográficas, morbilidades autorreferidas y percepción de salud, y tuvieron evaluado su estado nutricional. Para la presentación de los datos, se utilizaron frecuencias absolutas y relativas, así como intervalos de confianza del 95% (IC95%). Se realizaron ajustes en la regresión para verificar la asociación entre el estado nutricional (bajo peso o eutrofia, sobrepeso y obesidad) y la percepción de salud (buena o muy buena, y muy mala, mala o regular), y se utilizaron la razón de probabilidades (*odds ratio*, OR) y el IC95% como medidas de fuerza de asociación.

**Resultados:**

De los 3.264 participantes, 2.827 (88,0%) eran mujeres y 1.931 (56,6%) eran adultos; 1.352 (41,4%) presentaban sobrepeso y 944 (28,9%) obesidad; 1.730 (53,0%) reportaron hipertensión, 1.433 (44,3%) dislipidemia y 548 (16,8%) diabetes. En cuanto a la percepción de salud, 2.358 (72,3%) de los entrevistados la consideraron buena o muy buena. Se verificó que los individuos con obesidad tuvieron un 47,0% más de probabilidades de reportar una percepción de salud muy mala, mala o regular, en comparación con sus contrapartes.

**Conclusión:**

La obesidad se asoció con una peor percepción de la salud entre los participantes del Programa Academia de la Salud.

**Ethical aspects**

Research ethics committee: Universidade Federal de Minas Gerais. Certificate of submission for ethical appraisal: 0537.0.203.000-11. Approval date: 16/12/2011. Research ethics committee: Prefeitura de Belo Horizonte. Certificate of submission for ethical appraisal: 0537.0.203.410-11 A. Approval date: 22/12/2011. Informed consent record: Obtained from all participants prior to data collection.

## Introduction 

Obesity is characterized by excessive adiposity, resulting from a positive energy balance and social, cultural, and environmental factors ^1^. In 2020, it affected 988 million individuals worldwide, with projections reaching 1.9 billion by 2035 ^2^. In Brazil, the prevalence among adults in state capitals doubled from 2006 to 2024, increasing from 11.8% to 25.7% ^3^.

In addition to being a disease, obesity is a risk factor for noncommunicable chronic diseases, such as cardiovascular diseases, diabetes, hypertension, dyslipidemias, among others, affecting individuals' health and quality of life ^4^. Health perception is considered a simple, easily obtainable, and valid indicator for broadening the assessment of population health and well-being, as well as for monitoring clinical and public health outcomes, thereby contributing to the evaluation of the impact of noncommunicable chronic diseases on individuals' lives ^5^. 

Health perception is considered useful for identifying differences in health status among population subgroups defined by gender, race/ethnicity, social class, age, and other characteristics. When health perception is considered very poor, poor, or fair, it predicts morbidities, polypharmacy, mortality, functional decline, greater use of health services, and worsening quality of life ^5^. 

The association between nutritional status and health perception has also been investigated. A high body mass index was associated with poor health perception across 49 low- and middle-income countries ^6^. Health perception among individuals with obesity was similar to or worse than that of normal-weight individuals up to 10 years older and showed early health impairments resulting from obesity ^7^. 

In Belgium, in 2013, among 4,155 individuals, the likelihood of reporting very poor, poor, or fair health perception was 2.7 times higher among individuals with class I obesity, 3.7 times higher for class II obesity, and 5.9 times higher for class III obesity, demonstrating the influence of obesity severity on health perception ^8^. In Brazil, in 2019, individuals with overweight had 10% higher odds of very poor, poor, or fair health perception, while those with obesity had 38% higher odds, compared to those with normal weight ^9^. 

Health perception is therefore a powerful health indicator, with wide applicability in primary health care due to its ease of collection and predictive potential ^5^. Primary health care is the entry point to the health system and coordinates care within the Health Care Network, being responsible for health surveillance actions and the provision of comprehensive care. It is also responsible for developing health promotion, prevention, and control of conditions and diseases ^10^, including activities carried out under the Health Academy Program (*Programa Academia da Saúde*) ^11^. The Program is a health promotion and care service of the Brazilian Unified Health System (*Sistema Único de Saúde*, SUS), whose actions offered may positively influence health perception ^11^. 

This study aimed to analyze the association between nutritional status and health perception among participants of the Health Academy Program in Belo Horizonte, Minas Gerais, assuming an association between a higher prevalence of obesity and very poor, poor, or fair health perception. Based on the results, it is expected to support the development of obesity management actions that contribute to the health of Program participants and to the strengthening of this service in Brazilian primary health care ^5^^,^^11^. 

## Methods 

Study design

This is a cross-sectional study using baseline data from a randomized controlled community trial entitled "Consumption of fruits and vegetables in health promotion services in Belo Horizonte, Minas Gerais: associated factors and nutritional interventions" (*Consumo de frutas e hortaliças em serviços de promoção da saúde de Belo Horizonte, Minas Gerais: fatores associados e intervenções nutricionais*), which aimed to develop and evaluate an intervention to encourage fruit and vegetable consumption among participants of the Health Academy Program in Belo Horizonte, Minas Gerais ^12^.

Setting

The Health Academy Program was implemented in Belo Horizonte in 2006, primarily in territories with very high/high health vulnerability index ^13^, expanding in 2011 to Brazilian primary health care. The health vulnerability index synthesizes socioeconomic and sanitation variables and aims to guide public health policies and the allocation of municipal resources to reduce health inequities. Ranging from 0 to 1, higher values indicate greater vulnerability; values are classified as low, medium, high, or very high ^13^. 

At the municipal level, the Program offers regular physical exercise guided by a physical education professional, as well as health education and care activities, such as promoting adequate, healthy eating and reducing tobacco use, with the support of multidisciplinary teams. Admission occurs through spontaneous demand or referral from other SUS services ^14^.

Participants

Participants aged 20 years or older who had attended Health Academy Program activities in the previous month were included in the study at each Program unit. Pregnant women, individuals with cognitive impairments that prevented them from answering the questionnaire, and those who missed three scheduled data collection appointments were excluded ^12^.

Variables

The investigated variables were collected between 2013 and 2014. They included the following sociodemographic data: sex (female, male), age group (adult, older adult), educational level (up to 8 years, 9-11 years, 12 years or more of schooling), marital status (single, married, divorced/separated, widowed), and per capita income (up to the 50th percentile, above the 50th percentile). Per capita income was calculated by dividing the total monthly household income by the number of household residents. 

The variables age and educational level were categorized as adult and older adult, considering a cutoff point of 60 years for older adults, according to the Ministry of Health ^15^, and the educational level was categorized as up to 8 years, 9-11 years, and 12 years or more of schooling. Per capita income was categorized into two groups: up to the 50th percentile and above the 50th percentile, given the variable's non-normal distribution and a change in the minimum wage during the data collection period, which made a fixed cutoff point impossible.

Self-reported morbidities were investigated, such as hypertension (yes, no), dyslipidemia (yes, no), and diabetes (yes, no); medication use (yes, no); treatment for anxiety or mental illness (yes, no); smoking (yes, no); health perception (very poor, poor, fair, good, or very good); attempts to reduce weight (yes, no); and anthropometric measurements (weight and height). In the questionnaire, the health perception question was asked before the anthropometric measurements, thereby minimizing the risk of interviewer-induced bias. 

Participants' weight and height were measured using a digital scale and a portable stadiometer ^12^. Nutritional status was classified based on body mass index (body mass index=weight/height²), according to cutoff points proposed by the World Health Organization: underweight (<18.5 kg/m²), normal weight (≥18.5-24.9 kg/m²), overweight (≥25.0-29.9 kg/m²), class I obesity (≥30.0-34.9 kg/m²), class II obesity (≥35.0-39.9 kg/m²), and class III obesity (≥40.0 kg/m²) ^1^. Participants without weight and height data were excluded from the statistical analyses. 

For statistical analyses, health perception was reclassified as very poor, poor, or fair, and good or very good. Nutritional status was grouped into underweight/normal weight, overweight, and obese. 

Data sources and measurement

The questionnaire was developed based on national surveys, such as the Surveillance System for Risk and Protective Factors for Chronic Diseases by Telephone Survey (*Vigilância de Fatores de Risco e Proteção para Doenças Crônicas por Inquérito Telefônico*, Vigitel), the Household Budget Survey (*Pesquisa de Orçamentos Familiares*, POF), and the Household Survey on Risk Behaviors and Reported Morbidity for Noncommunicable Diseases (*Inquérito Domiciliar sobre Comportamento de Risco e Morbidade Referida de Doenças e Agravos Não Transmissíveis*), in addition to the research group's experience. To verify its adequacy for the target population, a pre-test and a pilot study were conducted in a Program unit not included in the research, followed by modifications to ensure participants' proper understanding ^12^. 

Bias

Data collection was carried out by a previously trained team responsible for conducting the interviews, supervised by the principal investigator and field supervisors. A field manual was used to address questions and standardize data collection ^12^.

Study size

The Health Academy Program units in this study were selected using a stratified simple random sampling design across the nine municipal administrative regions. The inclusion criteria were: operation in the morning; location in areas with medium, high, or very high health vulnerability index; no participation in food and nutrition research in the previous two years; and being operational in November 2012, when the sampling process was conducted. 

At that time, 50 Program units were operating in the municipality; six units located in areas with low health vulnerability index and two that had received intervention studies in the previous two years were excluded ^12^. Therefore, 42 Program units were eligible for the sampling process, and two units from each region were randomly selected and matched by health vulnerability index, resulting in 18 units being included. This sample was representative of the Program units in the municipality located in areas with medium and high/very high health vulnerability index, with 95% confidence and an error of less than 1.4% ^12^.

Statistical methods

Statistical analyses were performed using Stata/SE 14.2 software with a 5% significance level. 

Descriptive analysis of the overall sample and stratified by health perception included the calculation of absolute and relative frequencies and 95% confidence intervals (95%CI).

The sample was described according to health perception, and the chi-square test was used to assess the association between nutritional status and the outcome. In addition, the Bonferroni correction was applied to adjust p-values for multiple comparisons for variables with three or more categories (p-value after correction: 0.05/3=0.017 for educational level and nutritional status with three categories, p-value after correction: 0.05/6=0.008 for marital status, and p-value after correction: 0.05/10=0.005 for obesity severity with five categories). 

Logistic regression was adjusted for age, sex, marital status, educational level, per capita income, hypertension, dyslipidemia, diabetes, treatment for mental illness, medication use, smoking, quality of life, and attempts to reduce weight. These variables were selected based on previous studies that indicated their association with the exposure and the outcome ^5^^,^^9^^,^^16^^,^^17^. Because they showed a strong association with age (correlation coefficient>0.35), the variables educational level, hypertension, and medication use were excluded from the model. Therefore, the following variables were retained in the model as adjustment variables: age, sex, marital status, per capita income, dyslipidemia, diabetes, treatment for mental illness, smoking, quality of life, and attempt to reduce weight ^5^^,^^7^^,^^9^^,^^17^^,^^18^. 

The Hosmer-Lemeshow test was used to assess the goodness of fit of the final model, considering a p-value>0.050. As measures of association strength, odds ratios (OR) and corresponding 95% confidence intervals (95%CI) were used.

## Results 

A total of 3,763 individuals were identified in the Health Academy Program units, with 237 refusals (6.3%) and 112 exclusions (3.0%). The reasons for exclusion were: age under 20 years (n=23), three missed interview appointments (n=33), and cognitive difficulties (n=47). The reasons for the remaining nine exclusions were not recorded during data collection. A total of 3,414 (90.7%) participants were included. For this study, 150 individuals were excluded due to missing anthropometric data, totaling 3,264 participants. 

Of the participants, 2,872 (88.0%) were female, 1,931 (56.6%) were adults, 1,730 (53.0%) reported hypertension, 1,433 (44.3%) dyslipidemia, and 548 (16.8%) diabetes. Regarding nutritional status, 1,352 (41.4%) were overweight, and 944 (28.9%) had obesity. The prevalence of underweight in the sample was 20 (0.6%), and these participants were grouped with those of normal weight into the underweight or normal weight category (n=948; 29.7%). A total of 2,358 participants (72.3%) reported their health as good or very good (Table 1).

Individuals with very poor, poor, or fair health perception showed higher prevalence of older adults (51.2% versus 40.4%; p-value<0.001), lower prevalence of schooling between 9 and 11 years (20.7% vs. 32.8%; p-value<0.001) and 12 years or more (4.9% vs. 10.0%; p-value<0.001), lower prevalence of per capita income above the 50th percentile (41.2% vs. 50.9%; p-value<0.001), higher prevalence of hypertension (68.8% vs. 47.0%; p-value<0.001), dyslipidemia (55.7% vs. 40.0%; p-value<0.001), and diabetes (29.0% vs. 12.2%; p-value<0.001), lower prevalence of overweight (39.3% vs. 42.2%; p-value<0.001), and higher prevalence of obesity (36.8% vs. 25.9%; p-value<0.001), treatment for anxiety or mental health problems (29.1% vs. 15.7%; p-value<0.001), smoking (7.5% vs. 5.0%; p-value 0.005), medication use (89.9% vs. 72.2%; p-value<0.001), and attempts to reduce weight (65.0% vs. 59.2%; p-value 0.003), compared with those with good or very good health perception (Table 1). 

The prevalence of class I obesity was 688 (21.1%), class II was 196 (6.0%), and class III was 60 (1.8%). Individuals with very poor, poor, or fair health perception showed higher prevalence of class I obesity (24.5% vs. 19.7; p-value<0.001), class II obesity (8.3% vs. 5.1; p-value<0.001), and class III obesity (4.0% vs. 1.0; p-value<0.001) compared to those with good or very good health perception (Table 2). 

In the adjusted logistic regression, the odds of reporting very poor, poor, or fair health perception were 47.0% (95%CI 1.15; 1.89; p-value 0.002) higher among individuals with obesity compared to those with underweight or normal weight (Table 3). The Hosmer-Lemeshow test showed a p-value 0.524, indicating adequate model fit. When stratifying obesity by severity, participants with class II and class III obesity had 1.77 (95%CI 1.22; 2.58; p-value 0.003) and 4.05 (95%CI 2.24; 7.32; p-value<0.001) higher odds of reporting very poor, poor, or fair health perception compared to those with underweight or normal weight (Table 4). 

**Table 1:** Absolute and relative frequencies and 95% confidence intervals (95%CI) of the sociodemographic, health, and nutritional status characteristics of the overall sample, according to health perception. Health Academy Program (*Programa Academia da Saúde*). Belo Horizonte, 2013-2014 (n=3,264)

Variables	Participants (n=3,264; 100.0%)		Good or very good health perception (n=2,358; 72.3%)		Very poor, poor, or fair health perception (n=905; 27.7%)		p-value
	n	% (95%CI)	n	% (95%CI)	n	% (95%CI)	
Sex							0.205
Female	2,872	88.0 (86.8; 89.1)	2,086	88.5 (87.1; 89.7)	785	86.7 (84.4; 88.8)	
Male	392	12.0 (10.9; 13.2)	272	11.5 (10.3; 12.9)	120	13.3 (11.2; 15.6)	
Age group							<0.001
Adult	1,931	56.6 (54.9; 58.2)	1,459	59.6 (57.7; 61.6)	471	48.8 (45.6; 51.9)	
Older adult	1,483	43.4 (41.8; 45.1)	988	40.4 (38.4; 42.3)	495	51.2 (48.1; 54.4)	
Educational level (years of schooling)							<0.001
Up to 8 years	2,119	62.1 (60.5; 63.7)	1,401	57.2 (55.3; 59.2)	718	74.4 (71.5; 77.1)	
9-11 years	1,002	29.4 (27.9; 30.9)	802	32.8 (30.9; 34.7)	200	20.7 (18.3; 23.4)	
12 years or more	291	8.5 (7.6; 9.5)	244	10.0 (8.8; 11.2)	47	4.9 (3.7; 6.4)	
Marital status							0.070
Married	2,017	61.8 (60.1; 63.5)	1,479	62.7 (60.8; 64.7)	537	59.3 (56.1; 62.5)	
Divorced/separated	266	8.2 (7.3; 9.1)	189	8.0 (7.0; 9.2)	77	8.5 (6.9; 10.5)	
Single	459	14.1 (12.9; 15.3)	332	14.1 (12.7; 15.5)	127	14.0 (11.9; 16.5)	
Widowed	521	16.0 (14.7; 17.3)	358	15.2 (14.0; 16.7)	163	18.0 (15.6; 20.7)	
Per capita income							<0.001
Up to the 50th percentile	1,610	51.8 (50.0; 53.6)	1,104	49.1 (47.1; 51.2)	506	58.8 (55.4; 62.0)	
Above the 50th percentile	1,498	48.2 (46.4; 50.0)	1,143	50.9 (48.8; 52.9)	355	41.2 (38.0; 44.6)	
Hypertension	1,730	53.0 (51.3; 54.7)	1,107	47.0 (45.0; 49.0)	623	68.8 (65.7; 71.8)	<0.001
Dyslipidemia	1,433	44.3 (42.6; 46.0)	936	40.0 (38.0; 42.0)	497	55.7 (52.4; 59.0)	<0.001
Diabetes	548	16.8 (15.6; 18.4)	286	12.2 (10.9; 13.5)	262	29.0 (26.1; 32.0)	<0.001
Nutritional status							<0.001
With low weight or normal weight	968	29.7 (28.1; 31.3)	752	31.9 (30.0; 33.8)	216	23.9 (21.2; 26.8)	
Overweight	1,352	41.4 (39.7; 43.1)	996	42.2 (40.3; 44.3)	356	39.3 (36.2; 42.6)	
Obesity	944	28.9 (27.4; 30.5)	610	25.9 (24.1; 27.7)	333	36.8 (33.7; 40.0)	
Treatment for anxiety	632	19.4 (18.1; 20.8)	369	15.7 (14.2; 17.2)	263	29.1 (26.2; 32.1)	<0.001
Smoking	185	5.7 (4.9; 6.5)	117	5.0 (4.2; 5.9)	68	7.5 (6.0; 9.4)	0.003
Medication use	2,516	77.1 (75.7; 78.5)	1,702	72.2 (70.4; 74.0)	814	89.9 (87.8; 91.7)	<0.001
Attempt to reduce weight	1,983	60.8 (59.1; 62.5)	1,395	59.2 (57.2; 61.2)	688	65.0 (61.8; 68.0)	0.001

Missing data by variable: educational level=2; per capita income=304; hypertension=2; dyslipidemia=34; diabetes=7; body mass index=150; nutritional status=150; health perception=1; treatment for anxiety=2; smoking=1; medication use=5; and attempt to reduce weight=2.

**Table 2:** Absolute and relative frequencies and 95% confidence intervals (95%CI) of participants' nutritional status, according to health perception. Health Academy Program (*Programa Academia da Saúde*). Belo Horizonte, 2013-2014 (n=3,264)

Nutritional status	Participants (n=3,264)		Good or very good health perception (n=2,358)		Very poor, poor, or fair health perception (n=905)		p-value
	n	% (95%CI)	n	% (95%CI)	n	% (95%CI)	
With low weight or normal weight	968	29.7 (28.1; 31.3)	752	31.9 (30.0; 33.8)	216	23.9 (21.2; 26.8)	<0.001
Overweight	1,352	41.4 (39.7; 43.1)	996	42.2 (40.3; 44.3)	356	39.3 (36.2; 42.6)	
Class I obesity	688	21.1 (19.7; 22.5)	465	19.7 (18.2; 21.4)	222	24.5 (21.8; 27.5)	
Class II obesity	196	6.0 (5.2; 6.9)	121	5.1 (4.3 ;6.1)	75	8.3 (6.7; 10.3)	
Class III obesity	60	1.8 (1.4; 2.4)	24	1.0 (0.7; 1.5)	36	4.0 (2.9; 5.5)	

Missing data for nutritional status: 150.

**Table 3:** Unadjusted and adjusted odds ratios (OR) and 95% confidence intervals (95%CI) for the association between overweight and obesity and health perception. Health Academy Program (*Programa Academia da Saúde*). Belo Horizonte, 2013-2014 (n=3,263)

Nutritional status	Unadjusted OR (95%CI)	p-value	Adjusted OR (95%CI)	p-value
With low weight or normal weight	1.00	-	1.00	-
Overweight	1.24 (1.03; 1.51)	0.027	1.07 (0.86; 1.34)	0.534
Obesity	1.90 (1.55; 2.33)	<0.001	1.47 (1.15; 1.89)	0.002

**Table 4:** Unadjusted and adjusted odds ratios (OR) and 95% confidence intervals (95%CI) for the association between nutritional status and health perception. Health Academy Program (*Programa Academia da Saúde*). Belo Horizonte, 2013-2014 (n=3,263)

Nutritional status	Unadjusted OR (95%CI)	p-value	Adjusted OR (95%CI)	p-value
With low weight or normal weight	1.00		1.00	
Overweight	1.24 (1.03; 1.51)	0.027	1.07 (0.86; 1.34)	0.536
Class I obesity	1.66 (1.33; 2.07)	<0.001	1.28 (0.98; 1.67)	0.070
Class II obesity	2.16 (1.56; 2.99)	<0.001	1.77 (1.22; 2.58)	0.003
Class III obesity	5.22 (3.05; 8.95)	<0.001	4.05 (2.24; 7.32)	<0.001

## Discussion 

A high prevalence of good or very good health perception was identified among participants of the Health Academy Program, but health perception was negatively affected by obesity. 

Despite the useful findings of this study for actions within the Health Academy Program, its limitations must be considered. Participant selection may introduce bias, given that the sample comprises predominantly female users of a health promotion service, with a high proportion of older adults, potentially affecting the study's external validity ^19^. However, these are characteristics of users of health promotion services and of Brazilian primary health care. Moreover, the number of Program units is increasing in the country, and the results may help guide Program actions and justify its expansion ^11^. The findings may also apply to other primary health care settings in different regions of the country, considering similarities in the target population ^19^. 

Given the subjectivity of health perception, there may be inaccuracies arising from differences in interpretation and a lack of conceptual consensus. However, subjectivity may be considered positive, given its sensitivity to issues that are not objectively detectable ^20^. Self-reported morbidity may be underreported. Nevertheless, contact with health services, as in the population in this study, increases the likelihood of accurate reporting of health status ^21^. 

The same body mass index cutoff points were used for adults and older adults, without specificity for the elderly age group. However, this methodological choice allowed classification of obesity into grades and assessment of its association with health perception, an important finding of this study ^1^. 

The aggregation of health perception categories into good or very good and very poor, poor, or fair may also be considered a limitation, given that the fair category is not necessarily negative. However, several studies have adopted this grouping strategy, treating the fair category as a negative outcome, thereby facilitating comparisons across studies ^5^^,^^18^. It is also important to mention, as an inherent limitation of the cross-sectional design, the inability to infer causality. 

The prevalence of obesity in this study, approximately 29% (95%CI 27.4; 30.5), was higher than the national prevalence of approximately 18% and the prevalence observed in Belo Horizonte, between 15% and 17%, during the data collection period in 2013 and 2014 ^3^. This finding may be due to the use of the Health Academy Program, given the prior presence of obesity, either through spontaneous demand or referral from the health care network ^14^. The main reason for seeking the service was health improvement, reported as 45% in Recife ^22^, 47% in Florianópolis ^23^, and 75% among women in Aracaju ^24^. 

The search for the program for weight reduction is justified by the provision of supervised physical exercise and actions promoting adequate and healthy eating, with lifestyle modification being one of the main recommendations for obesity management ^11^. Therefore, the program provides a strategic setting to support health care networks in treating obesity ^11^. 

Most individuals reported good or very good health perception, with values similar to or higher than those observed in other studies, ranging from 46% to 72% ^9^^,^^17^^,^^18^^,^^25^. By offering health promotion activities, the program may favor a better health perception, contributing to improvements in quality of life, physical fitness, social interaction, self-esteem, diet, reductions in insomnia and stress, medication use, weight, body mass index, and control of hypertension and diabetes ^11^. The program's presence in Belo Horizonte since 2006 has made it well established in the municipality, potentially influencing adherence and health outcomes. Belo Horizonte has been described as having an efficient primary health care system, which may also contribute to the findings ^26^. 

Despite the high prevalence of good or very good health perception, individuals with obesity had 47% higher odds of reporting very poor, poor, or fair health perception. In 2008, individuals with obesity had 26% higher odds (95%CI 1.07; 1.49) of reporting fair or poor health perception compared to those with normal weight ^18^. This highlights the health burden that obesity imposes on individuals' lives ^4^. 

Obesity is associated with a higher prevalence of noncommunicable chronic diseases, decline in quality of life, reduced physical functioning, physical limitations, pain, and reduced vitality, affecting individuals' ability to engage in daily activities ^4^. It also increases body image dissatisfaction, social isolation, discrimination, depression, and anxiety ^4^. 

The stigma experienced by individuals with obesity is associated with physical inactivity, unhealthy eating behaviors, weight gain, and difficulties in accessing health services, exacerbating health-related consequences ^4^. Such effects may be mitigated in the long term through weight reduction. In Sweden, from 2002 to 2014, among 2,816 individuals aged 30 to 75 years, a higher baseline body mass index predicted a higher likelihood of very poor, poor, or fair health perception ten years later among those who maintained or gained weight ^16^. 

Despite the expansion of the Health Academy Program, some obesity management actions in primary health care remain restricted to primary health care units. However, these units show limited adequacy for effective obesity management, with limitations in physical infrastructure, multidisciplinary teams, and user registration systems, among others ^27^. Professional qualifications are another limiting factor, with limited interdisciplinary support, diagnostic difficulties, and challenges in applying collective approaches to obesity management being important barriers ^28^. 

The prevalence of class II and III obesity was approximately 6% and 2%, respectively. Individuals with class II and III obesity reported difficulties in accessing treatment within the SUS, including limited resource availability and insufficiently prepared health teams for obesity management ^29^. The Health Academy Program may not reach this population, given that entry into the service occurs in part through referrals from primary health care ^14^.

Comprehensive follow-up of individuals with obesity within the SUS is essential, considering the presence of other noncommunicable chronic diseases, the progressive worsening of obesity, and its associations with negative health outcomes ^4^. Given the sensitivity of health perception to subjective aspects, its use in longitudinal evaluations of individuals with obesity in primary health care may contribute to the development and assessment of health promotion, prevention, and recovery actions aimed at comprehensive care and quality of life for the population with obesity ^5^. The expansion and strengthening of the Health Academy Program in the country, and greater integration with health care networks, are essential to reduce access inequalities and improve population health, including among individuals with obesity ^11^.

Strengths of this study include the sample size, methodological rigor, data quality control, and internal validity of the findings. 

Based on these results, it was concluded that participants in the Health Academy Program had a high prevalence of good or very good health perception; however, obesity was associated with higher odds of very poor, poor, or fair health perception. 

## Data Availability

The contents of the database are available upon request.
