# Supplementary material for: Association between nutritional status and health perception among participants of the Academida da Saúde Program: a cross-sectional study, Belo Horizonte, 2013-2014
Source: Epidemiol Serv Saude. 2026 Jul 18;35:e20250992. doi: 10.1590/S2237-96222026v35e20250992.en (PMC13686295; doi:10.1590/S2237-96222026v35e20250992.en)
Supplement: Translation

# Translation: Associação entre estado nutricional e percepção de saúde em participantes do Programa Academia da Saúde: estudo transversal, Belo Horizonte, 2013-2014

**DOI:** 10.1590/S2237-96222026v35e20250992.pt

**Authors:** Tatiane Moreira Alves Garcia, Maria Cecília Ramos de Carvalho, Aline Cristine Souza Lopes

**Affiliations:** 1 Universidade Federal de Minas Gerais, Belo Horizonte, MG, Brasil Universidade Federal de Minas Gerais Belo Horizonte MG Brasil

**Keywords:** Autoavaliação diagnóstica, Obesidade, Promoção da Saúde, Atenção Primária à Saúde, Estudos Transversais

## Abstract

**Objetivo:**

Analisar a associação entre estado nutricional e percepção de saúde entre participantes do Programa Academia da Saúde de Belo Horizonte, Minas Gerais.

**Métodos:**

Estudo transversal com dados da linha de base de ensaio comunitário controlado randomizado conduzido em amostra representativa do Programa. Indivíduos com 20 anos ou mais responderam questionário contendo variáveis sociodemográficas, morbidades referidas e percepção de saúde, e tiveram o estado nutricional aferido. Para apresentação dos dados, utilizaram-se frequências absoluta e relativa e intervalos de confiança de 95% (IC95%). Utilizaram-se ajustes na regressão para verificar a associação entre o estado nutricional (com baixo peso ou eutrofia, sobrepeso e obesidade) e a percepção de saúde (boa ou muito boa e muito ruim, ruim ou regular) e razão de chances (*odds ratio*) e IC95% como medida de força de associação.

**Resultados:**

Dos 3.264 participantes, 2.827 (88,0%) eram mulheres e 1.931 (56,6%) adultos; 1.352 (41,4%) apresentavam sobrepeso e 944 (28,9%) obesidade; 1.730 (53,0%) relataram hipertensão, 1.433 (44,3%) dislipidemia e 548 (16,8%) diabetes. Quanto à percepção de saúde, 2.358 (72,3%) entrevistados consideraram sua saúde como boa ou muito boa. Verificou-se que indivíduos com obesidade tiveram 47,0% mais chances de relatar percepção de saúde muito ruim, ruim ou regular, comparados às contrapartes.

**Conclusão:**

A obesidade foi associada à pior percepção da saúde entre participantes do Programa Academia da Saúde.

**Aspectos éticos**

Comitê de ética em pesquisa: Universidade Federal de Minas Gerais. Certificado de apresentação de apreciação ética: 0537.0.203.000-11. Data de aprovação: 16/12/2011. Comitê de ética em pesquisa: Prefeitura de Belo Horizonte. Certificado de apresentação de apreciação ética: 0537.0.203.410-11 A. Data de aprovação: 22/12/2011. Registro do consentimento livre e esclarecido: Obtido de todos os participantes antes da coleta de dados.

## Introdução 

A obesidade caracteriza-se pela adiposidade excessiva, decorrente de balanço energético positivo, fatores sociais, culturais e ambientais ^1^. Em 2020, atingiu 988 milhões de indivíduos mundialmente, projetando-se alcance de 1,9 bilhão em 2035 ^2^. No Brasil, a prevalência entre adultos das capitais dobrou de 2006 a 2024, passando de 11,8% para 25,7% ^3^.

Além de doença, obesidade é fator de risco para doenças crônicas não transmissíveis, como cardiovasculares, diabetes, hipertensão, dislipidemias, entre outras, afetando saúde e qualidade de vida dos indivíduos ^4^. Considera-se a percepção de saúde um indicador simples, de fácil obtenção e válido para ampliar a avaliação da saúde e bem-estar populacional, que monitora também resultados clínicos e de saúde pública, contribuindo para avaliar o impacto das doenças crônicas não transmissíveis na vida dos indivíduos ^5^. 

Considera-se a percepção de saúde útil para identificar diferenças no estado de saúde entre subgrupos da população, definidos por gênero, raça/etnia, classe social, idade, entre outras características. Quando a percepção de saúde é considerada muito ruim, ruim ou regular, prediz morbidades, polifarmácia, mortalidade, declínio funcional, maior utilização de serviços de saúde e piora da qualidade de vida ^5^. 

Também se investiga a associação entre estado nutricional e percepção de saúde. O índice de massa corporal elevado associou-se à percepção de saúde ruim em 49 países de baixa e média renda ^6^. A percepção de saúde entre indivíduos com obesidade foi semelhante ou pior que a de indivíduos eutróficos até 10 anos mais velhos e evidenciou prejuízos precoces à saúde decorrentes da obesidade ^7^. 

Na Bélgica, em 2013, entre 4.155 indivíduos, a chance de apresentar percepção de saúde muito ruim, ruim ou regular era 2,7 vezes maior entre indivíduos com obesidade grau I, 3,7 vezes maior para obesidade grau II, e 5,9 vezes maior para obesidade grau III, o que evidenciou a influência do grau de obesidade na percepção de saúde ^8^. No Brasil, em 2019, indivíduos com sobrepeso apresentaram chances 10% maiores de percepção de saúde muito ruim, ruim ou regular, enquanto aqueles com obesidade apresentaram chances 38% maiores, comparados àqueles em eutrofia ^9^. 

A percepção de saúde constitui, portanto, potente indicador de saúde, com grande aplicabilidade na atenção primária à saúde por sua fácil obtenção e potencial preditor ^5^. A atenção primária à saúde é porta de entrada para o sistema de saúde e coordenadora do cuidado na Rede de Atenção à Saúde, responsabilizando-se pelas ações de vigilância à saúde e oferta do cuidado integral. Também se responsabiliza pelo desenvolvimento de ações de promoção da saúde, prevenção e controle de agravos e doenças ^10^, incluindo aquelas realizadas no âmbito do Programa Academia da Saúde ^11^. O Programa é um serviço de promoção e cuidado à saúde do Sistema Único de Saúde (SUS), cujas ações ofertadas podem refletir positivamente sobre a percepção de saúde ^11^. 

Este trabalho objetivou analisar a associação entre estado nutricional e percepção de saúde entre participantes do Programa Academia da Saúde de Belo Horizonte, Minas Gerais, hipotetizando uma associação entre maior prevalência de obesidade e percepção de saúde muito ruim, ruim ou regular. Partindo dos resultados, espera-se subsidiar o desenvolvimento de ações de manejo da obesidade que contribuam para a saúde dos participantes do Programa e para o fortalecimento desse serviço na atenção primária à saúde brasileira ^5^^,^^11^. 

## Métodos 

Delineamento do estudo

Trata-se de estudo transversal com dados da linha de base de ensaio comunitário controlado randomizado denominado "Consumo de frutas e hortaliças em serviços de promoção da saúde de Belo Horizonte, Minas Gerais: fatores associados e intervenções nutricionais", que objetivou desenvolver e avaliar a intervenção de incentivo ao consumo de frutas e hortaliças entre os participantes do Programa Academia da Saúde de Belo Horizonte, Minas Gerais ^12^.

Contexto

Implementou-se o Programa Academia da Saúde em Belo Horizonte em 2006, em territórios com índice de vulnerabilidade à saúde muito elevado/elevado, prioritariamente ^13^, expandindo-se, em 2011, para a atenção primária à saúde brasileira. O índice de vulnerabilidade à saúde sintetiza variáveis socioeconômicas e de saneamento e visa direcionar políticas públicas de saúde e alocação de recursos municipais, objetivando reduzir iniquidades em saúde. Variando de 0 a 1, quanto maior o valor, mais vulnerável o território, classificado em: baixo, médio, elevado e muito elevado ^13^. 

Municipalmente, o Programa oferta a prática regular de exercícios físicos orientada por profissional de educação física e atividades de educação e cuidado da saúde, como a promoção da alimentação adequada e saudável e o controle do tabagismo, com o apoio das equipes multiprofissionais. O ingresso é por demanda espontânea ou referenciamento por outros serviços do SUS ^14^.

Participantes

Foram incluídos no estudo, em cada unidade do Programa Academia da Saúde, participantes com 20 anos ou mais e frequentes às atividades do programa no mês anterior. Excluíram-se mulheres gestantes, pessoas com dificuldades cognitivas que impedissem responder ao questionário e indivíduos que faltaram a três agendamentos da coleta de dados ^12^.

Variáveis

Coletaram-se as variáveis investigadas entre 2013 e 2014, que constaram dos seguintes dados sociodemográficos: sexo (feminino, masculino), faixa etária (adulto, idoso), escolaridade (até 8 anos, 9-11 anos, 12 anos ou mais), estado civil (solteiro, casado, divorciado/separado, viúvo), renda per capita (até o percentil 50, acima do percentil 50). Calculou-se a renda per capita dividindo-se a renda familiar mensal pelo total de moradores do domicílio. 

Categorizaram-se as variáveis idade em adulto e idoso, considerando ponto de corte de 60 anos para idosos, de acordo com o Ministério da Saúde ^15^, e escolaridade em até 8 anos, 9-11 anos e 12 anos ou mais. A renda per capita foi categorizada em até o percentil 50 e acima do percentil 50, considerando a distribuição não normal da variável e uma mudança no valor do salário mínimo durante a coleta de dados, impossibilitando a adoção de um ponto de corte fixo.

Investigaram-se o autorrelato de morbidades referidas, como hipertensão (sim, não), dislipidemia (sim, não) e diabetes (sim, não); o uso de medicamentos (sim, não); o tratamento para nervosismo ou doença mental (sim, não); o tabagismo (sim, não); a percepção de saúde (muito ruim, ruim, regular, boa ou muito boa); as tentativas de redução de peso (sim, não); e a aferição das medidas antropométricas (peso e estatura). No questionário, a pergunta sobre a percepção de saúde foi realizada antes das medidas antropométricas, o que minimizou o risco de viés induzido pelo entrevistador. 

Aferiram-se peso e altura dos participantes em balança digital e estadiômetro portátil ^12^. Classificou-se o estado nutricional partindo do índice de massa corporal (índice de massa corporal=peso/altura²), segundo pontos de corte propostos pela Organização Mundial de Saúde: baixo peso (<18,5 kg/m²), eutrofia (≥18,5-24,9 kg/m²), sobrepeso (≥25,0-29,9 kg/m²), obesidade grau I (≥30,0-34,9 kg/m²), obesidade grau II (≥35,0-39,9 kg/m²) e obesidade grau III (≥40,0 kg/m²) ^1^. Excluíram-se das análises estatísticas participantes sem dados de peso e altura. 

Para as análises estatísticas, reclassificou-se a percepção de saúde como muito ruim, ruim ou regular e boa ou muito boa. O estado nutricional foi agrupado em com baixo peso ou eutrofia, sobrepeso e obesidade. 

Fonte de dados e mensuração

Desenvolveu-se o questionário partindo de inquéritos nacionais, como o Sistema de Vigilância de Fatores de Risco e Proteção para Doenças Crônicas por Inquérito Telefônico, Pesquisa de Orçamentos Familiares e Inquérito Domiciliar sobre Comportamento de Risco e Morbidade Referida de Doenças e Agravos Não Transmissíveis, além de experiência do grupo de pesquisa. Para verificar sua adequação ao público, procederam-se pré-teste e estudo-piloto em uma unidade do Programa não participante da pesquisa, com posteriores modificações para a adequada compreensão dos participantes ^12^. 

Viés

Realizou-se a coleta de dados por equipe previamente treinada para a condução das entrevistas, supervisionada pela pesquisadora responsável e por supervisores de campo. Utilizou-se manual de campo visando esclarecer dúvidas e padronizar a coleta dos dados ^12^.

Tamanho do estudo

As unidades do Programa Academia da Saúde neste estudo foram obtidas por amostra aleatória simples estratificada pelas nove regionais administrativas municipais. Os critérios de inclusão foram: funcionamento pela manhã; localização em área de índice de vulnerabilidade à saúde médio, elevado ou muito elevado; não participação em pesquisas sobre alimentação e nutrição nos dois últimos anos; e funcionamento em novembro de 2012, quando foi realizado o processo amostral. 

À época, eram 50 unidades do Programa em funcionamento no município, excluindo-se seis unidades localizadas em áreas com índice de vulnerabilidade à saúde baixo, e duas que haviam recebido estudos de intervenção nos dois anos anteriores ^12^. Assim, 42 unidades do Programa foram elegíveis para o processo amostral, sorteando-se duas unidades de cada regional, pareadas por índice de vulnerabilidade à saúde, o que resultou em 18 unidades incluídas. Essa amostra foi representativa das unidades do Programa do município localizadas em áreas de médio e elevado/muito elevado índice de vulnerabilidade à saúde, com 95% de confiança e erro menor que 1,4% ^12^.

Métodos estatísticos

As análises estatísticas foram realizadas com o software Stata/SE 14.2, adotando-se o nível de significância de 5%. 

A análise descritiva da amostra geral e estratificada pela percepção de saúde constou do cálculo das frequências absolutas e relativas e intervalos de confiança de 95% (IC95%).

Foram realizados a descrição da amostra estratificada pela percepção de saúde e o teste qui-quadrado para verificar associação entre o estado nutricional e o desfecho, além da correção de Bonferroni para ajustar o p-valor em comparações múltiplas para variáveis com três categorias ou mais (p corrigido: 0,05/3=0,017 para escolaridade e estado nutricional com três categorias, p corrigido: 0,05/6=0,008 para estado civil, e p corrigido: 0,05/10=0,005 para graus de obesidade com cinco categorias). 

Ajustou-se a regressão logística por idade, sexo, estado civil, escolaridade, renda per capita, hipertensão, dislipidemia, diabetes, tratamento para doença mental, uso de medicamentos, tabagismo, qualidade de vida e tentativa de redução do peso. Tais variáveis foram selecionadas conforme estudos prévios que indicaram sua associação com a exposição e o desfecho ^5^^,^^9^^,^^16^^,^^17^. Por apresentarem forte associação com a idade (coeficientes de correlação>0,35), as variáveis escolaridade, hipertensão e uso de medicamentos foram excluídas do modelo. Portanto, foram mantidas no modelo como variáveis de ajuste: idade, sexo, estado civil, renda per capita, dislipidemia, diabetes, tratamento para doença mental, tabagismo, qualidade de vida e tentativa de redução do peso ^5^^,^^7^^,^^9^^,^^17^^,^^18^. 

Utilizou-se o teste Hosmer-Lemeshow para verificar o ajuste do modelo final, considerando p-valor>0,050. Como medida de força de associação, utilizaram-se a razão de chances (*odds ratio*, OR) e o respectivo IC95%.

## Resultados

Identificaram-se 3.763 indivíduos nas unidades do Programa Academia da Saúde, sendo contabilizadas 237 recusas (6,3%) e 112 exclusões (3,0%). Os motivos das exclusões foram: idade menor que 20 anos (n=23), três faltas no agendamento da entrevista (n=33) e dificuldades cognitivas (n=47). Os motivos das nove exclusões restantes não foram registrados durante a coleta dos dados. Totalizaram-se 3.414 (90,7%) participantes. Para este estudo, foram excluídos 150 indivíduos pela ausência de dados antropométricos, o que totalizou 3.264 participantes. 

Dos participantes, 2.872 (88,0%) eram do sexo feminino, 1.931 (56,6%) adultos, 1.730 (53,0%) relataram hipertensão, 1.433 (44,3%) dislipidemias e 548 (16,8%) diabetes. Quanto ao estado nutricional, 1.352 (41,4%) possuíam sobrepeso, e 944 (28,9%) obesidade. A prevalência de baixo peso na amostra foi de 20 (0,6%), sendo esses participantes agrupados com aqueles em eutrofia na categoria baixo peso ou eutrofia (n=948; 29,7%). Consideraram sua saúde boa ou muito boa 2.358 (72,3%) participantes (Tabela 1).

Os indivíduos com percepção de saúde muito ruim, ruim ou regular apresentaram maiores prevalências de idosos (51,2% versus 40,4%; p-valor<0,001; menores prevalências de escolaridade entre 9 e 11 anos (20,7% vs. 32,8%; p-valor<0,001) e igual ou superior a 12 anos (4,9% vs. 10,0%; p-valor<0,001); menores prevalências de renda per capita acima do percentil 50 (41,2% vs. 50,9%; p-valor<0,001); maiores prevalências de hipertensão (68,8% vs. 47,0%; p-valor<0,001), dislipidemia (55,7% vs. 40,0%; p-valor<0,001) e diabetes (29,0% vs. 12,2%; p-valor<0,001); menor prevalência de sobrepeso (39,3% vs. 42,2%; p-valor<0,001); e maior de obesidade (36,8% vs. 25,9%; p-valor<0,001), tratamento para nervosismo (29,1% vs. 15,7%; p-valor<0,001), tabagismo (7,5% vs. 5,0%; p-valor 0,005), uso de medicamentos (89,9% vs. 72,2%; p-valor<0,001) e tentativas de redução de peso (65,0% vs. 59,2%; p-valor 0,003), comparados com aqueles com percepção de saúde boa ou muito boa (Tabela 1). 

A prevalência de obesidade grau I foi de 688 (21,1%), grau II de 196 (6,0%) e de grau III 60 (1,8%). Indivíduos com percepção de saúde muito ruim, ruim ou regular apresentaram maiores prevalências de obesidade grau I (24,5% vs. 19,7; p-valor<0,001), grau II (8,3% vs. 5,1; p-valor<0,001) e grau III (4,0% vs. 1,0; p-valor<0,001) comparados àqueles com percepção de saúde boa ou muito boa (Tabela 2). 

Na regressão logística ajustada, a chance de relatar percepção de saúde muito ruim, ruim ou regular foi 47,0% (IC95% 1,15; 1,89; p-valor 0,002) superior entre indivíduos com obesidade, quando comparados àqueles com baixo peso ou eutrofia (Tabela 3). O teste de Hosmer-Lemeshow apresentou p-valor 0,524, o que indicou ajuste adequado do modelo. Ao se estratificar a obesidade em graus, participantes com obesidade graus II ou III apresentaram 1,77 (IC95% 1,22; 2,58; p-valor 0,003) e 4,05 (IC95% 2,24; 7,32; p-valor<0,001) mais chances de relatar percepção de saúde muito ruim, ruim ou regular comparados àqueles com baixo peso ou eutrofia (Tabela 4). 

**Tabela 1:** Frequência absoluta e relativa e intervalo de confiança de 95% (IC95%) das características sociodemográficas, de saúde e estado nutricional da amostra completa, segundo a percepção de saúde. Programa Academia da Saúde. Belo Horizonte, 2013-2014 (n=3.264)

Variáveis	Participantes (n=3.264; 100,0%)		Percepção de saúde boa ou muito boa (n=2.358; 72,3%)		Percepção de saúde muito ruim, ruim ou regular (n=905; 27,7%)		p-valor
	n	% (IC95%)	n	% (IC95%)	n	% (IC95%)	
Sexo							0,205
Feminino	2.872	88,0 (86,8; 89,1)	2.086	88,5 (87,1; 89,7)	785	86,7 (84,4; 88,8)	
Masculino	392	12,0 (10,9; 13,2)	272	11,5 (10,3; 12,9)	120	13,3 (11,2; 15,6)	
Faixa etária							<0,001
Adulto	1.931	56,6 (54,9; 58,2)	1.459	59,6 (57,7; 61,6)	471	48,8 (45,6; 51,9)	
Idoso	1.483	43,4 (41,8; 45,1)	988	40,4 (38,4; 42,3)	495	51,2 (48,1; 54,4)	
Escolaridade							<0,001
Até 8 anos	2.119	62,1 (60,5; 63,7)	1.401	57,2 (55,3; 59,2)	718	74,4 (71,5; 77,1)	
9-11 anos	1.002	29,4 (27,9; 30,9)	802	32,8 (30,9; 34,7)	200	20,7 (18,3; 23,4)	
12 anos ou mais	291	8,5 (7,6; 9,5)	244	10,0 (8,8; 11,2)	47	4,9 (3,7; 6,4)	
Estado civil							0,070
Casado	2.017	61,8 (60,1; 63,5)	1.479	62,7 (60,8; 64,7)	537	59,3 (56,1; 62,5)	
Divorciado/separado	266	8,2 (7,3; 9,1)	189	8,0 (7,0; 9,2)	77	8,5 (6,9; 10,5)	
Solteiro	459	14,1 (12,9; 15,3)	332	14,1 (12,7; 15,5)	127	14,0 (11,9; 16,5)	
Viúvo	521	16,0 (14,7; 17,3)	358	15,2 (14,0; 16,7)	163	18,0 (15,6; 20,7)	
Renda per capita							<0,001
Até o percentil 50	1.610	51,8 (50,0; 53,6)	1.104	49,1 (47,1; 51,2)	506	58,8 (55,4; 62,0)	
Acima do percentil 50	1.498	48,2 (46,4; 50,0)	1,143	50,9 (48,8; 52,9)	355	41,2 (38,0; 44,6)	
Hipertensão	1.730	53,0 (51,3; 54,7)	1.107	47,0 (45,0; 49,0)	623	68,8 (65,7; 71,8)	<0,001
Dislipidemia	1.433	44,3 (42,6; 46,0)	936	40,0 (38,0; 42,0)	497	55,7 (52,4; 59,0)	<0,001
Diabetes	548	16,8 (15,6; 18,4)	286	12,2 (10,9; 13,5)	262	29,0 (26,1; 32,0)	<0,001
Estado nutricional							<0,001
Com baixo peso ou eutrofia	968	29,7 (28,1; 31,3)	752	31,9 (30,0; 33,8)	216	23,9 (21,2; 26,8)	
Sobrepeso	1.352	41,4 (39,7; 43,1)	996	42,2 (40,3; 44,3)	356	39,3 (36,2; 42,6)	
Obesidade	944	28,9 (27,4; 30,5)	610	25,9 (24,1; 27,7)	333	36,8 (33,7; 40,0)	
Tratamento para nervosismo	632	19,4 (18,1; 20,8)	369	15,7 (14,2; 17,2)	263	29,1 (26,2; 32,1)	<0,001
Tabagismo	185	5,7 (4,9; 6,5)	117	5,0 (4,2; 5,9)	68	7,5 (6,0; 9,4)	0,003
Uso de medicamentos	2.516	77,1 (75,7; 78,5)	1.702	72,2 (70,4; 74,0)	814	89,9 (87,8; 91,7)	<0,001
Tentativa de redução de peso	1.983	60,8 (59,1; 62,5)	1.395	59,2 (57,2; 61,2)	688	65,0 (61,8; 68,0)	0,001

Dados faltantes por variável: escolaridade=2; renda per capita=304; hipertensão=2; dislipidemia=34; diabetes=7; índice de massa corporal=150; estado nutricional=150; percepção de saúde=1; tratamento para nervosismo=2; tabagismo=1; uso de medicamentos=5; e tentativa de redução de peso=2.

**Tabela 2:** Frequência absoluta e relativa e intervalo de confiança de 95% (IC95%) do estado nutricional dos participantes, segundo a percepção de saúde. Programa Academia da Saúde. Belo Horizonte, 2013-2014 (n=3.264)

Estado nutricional	Participantes (n=3.264)		Percepção de saúde boa ou muito boa (n=2.358)		Percepção de saúde muito ruim, ruim ou regular (n=905)		p-valor
	n	% (IC95%)	n	% (IC95%)	n	% (IC95%)	
Com baixo peso ou eutrofia	968	29,7 (28,1; 31,3)	752	31,9 (30,0; 33,8)	216	23,9 (21,2; 26,8)	<0,001
Sobrepeso	1.352	41,4 (39,7; 43,1)	996	42,2 (40,3; 44,3)	356	39,3 (36,2; 42,6)	
Obesidade grau I	688	21,1 (19,7; 22,5)	465	19,7 (18,2; 21,4)	222	24,5 (21,8; 27,5)	
Obesidade grau II	196	6,0 (5,2; 6,9)	121	5,1 (4,3 ;6,1)	75	8,3 (6,7; 10,3)	
Obesidade grau III	60	1,8 (1,4; 2,4)	24	1,0 (0,7; 1,5)	36	4,0 (2,9; 5,5)	

Dados faltantes do estado nutricional: 150.

**Tabela 3:** Razão de chances (*odds ratio*, OR) bruta e ajustada e intervalo de confiança de 95% (IC95%) da associação entre sobrepeso e obesidade e percepção de saúde. Programa Academia da Saúde. Belo Horizonte, 2013-2014 (n=3.263)

Estado nutricional	OR não ajustada (IC95%)	p-valor	OR ajustada (IC95%)	p-valor
Com baixo peso ou eutrofia	1,00		1,00	
Sobrepeso	1,24 (1,03; 1,51)	0,027	1,07 (0,86; 1,34)	0,534
Obesidade	1,90 (1,55; 2,33)	<0,001	1,47 (1,15; 1,89)	0,002

**Tabela 4:** Razão de chances (*odds ratio*, OR) bruta e ajustada e intervalo de confiança de 95% (IC95%) da associação entre estado nutricional e percepção de saúde. Programa Academia da Saúde. Belo Horizonte, 2013-2014 (n=3.263)

Estado nutricional	OR não ajustada (IC95%)	p-valor	OR ajustada (IC95%)	p-valor
Com baixo peso ou eutrofia	1,00		1,00	
Sobrepeso	1,24 (1,03; 1,51)	0,027	1,07 (0,86; 1,34)	0,536
Obesidade grau I	1,66 (1,33; 2,07)	<0,001	1,28 (0,98; 1,67)	0,070
Obesidade grau II	2,16 (1,56; 2,99)	<0,001	1,77 (1,22; 2,58)	0,003
Obesidade grau III	5,22 (3,05; 8,95)	<0,001	4,05 (2,24; 7,32)	<0,001

## Discussão 

Identificou-se elevada prevalência de percepção de saúde boa ou muito boa entre participantes do Programa Academia da Saúde, mas a percepção de saúde foi afetada negativamente pela obesidade. 

Apesar dos resultados úteis deste trabalho para ações do Programa Academia da Saúde, suas limitações precisam ser ponderadas. A seleção dos participantes pode ser fonte de viés, considerando o público composto por participantes de serviço de promoção da saúde, predominantemente feminino, com alta prevalência de idosos, o que pode afetar a validade externa do estudo ^19^. Entretanto, trata-se das características do público de serviços de promoção da saúde e da atenção primária à saúde brasileira. Ademais, o número de unidades do Programa cresce no país, podendo os resultados contribuir para delinear ações do Programa e justificar sua expansão ^11^. Os resultados também podem ser aplicáveis a outros cenários da atenção primária à saúde em diferentes regiões do país, considerando semelhanças do público ^19^. 

Levando em conta a subjetividade da percepção de saúde, pode haver imprecisões devido a diferenças nas interpretações e na inexistência de consenso conceitual. Entretanto, a subjetividade pode ser positiva, considerando a sensibilidade para questões indetectáveis objetivamente ^20^. O relato de morbidades pode ser subnotificado. Contudo, estar em serviços de saúde, como o público desta pesquisa, aumenta a possibilidade de relatar corretamente o estado de saúde ^21^. 

Utilizaram-se os mesmos pontos de corte de índice de massa corporal para indivíduos adultos e idosos, inespecificamente para a faixa etária idosa. Entretanto, essa escolha metodológica permitiu obter a classificação da obesidade em graus e verificar sua associação com a percepção de saúde, importante resultado deste estudo ^1^. 

A agregação das categorias da percepção de saúde em boa ou muito boa e muito ruim, ruim ou regular também pode ser uma limitação, considerando que não necessariamente a categoria regular é negativa. Contudo, diversos estudos utilizam essa agregação, considerando a categoria regular como negativa, possibilitando a comparabilidade dos resultados ^5^^,^^18^. Também é importante citar como limitação inerente ao delineamento transversal a impossibilidade de estabelecer relação causal. 

A prevalência de obesidade neste estudo, de aproximadamente 29% (IC95% 27,4; 30,5), foi superior às prevalências nacionais, de aproximadamente 18%, e em Belo Horizonte,entre 15% e 17%, no período da coleta, em 2013 e 2014 ^3^. Esse resultado possivelmente deriva da procura pelo Programa Academia da Saúde devido à presença prévia da obesidade, seja por demanda espontânea, seja por encaminhamento da rede de atenção à saúde ^14^. O principal motivo pela busca do serviço foi a melhoria da saúde, sendo 45% em Recife ^22^, 47% em Florianópolis ^23^ e 75% entre mulheres de Aracaju ^24^. 

A busca do programa para redução de peso justifica-se pela oferta de exercício físico orientado e de ações de promoção da alimentação adequada e saudável, sendo a mudança de modos de vida uma das principais orientações para tratar a obesidade ^11^. Assim, o programa constitui cenário estratégico para apoiar as redes de atenção à saúde no tratamento da obesidade ^11^. 

A maioria dos indivíduos relatou percepção de saúde boa ou muito boa, com valores semelhantes ou superiores a outros estudos, o que variou entre 46% e 72% ^9^^,^^17^^,^^18^^,^^25^. Ao ofertar atividades de promoção da saúde, o programa pode favorecer melhor percepção de saúde, contribuindo para melhoria da qualidade de vida, aptidão física, interação social, autoestima, alimentação, redução da insônia, estresse, uso de medicamentos, peso, índice de massa corporal, e controle da hipertensão e diabetes ^11^. A presença do programa em Belo Horizonte desde 2006 o torna bem consolidado no município, influenciando possivelmente a adesão e resultados em saúde. Belo Horizonte apresentou atenção primária à saúde considerada eficiente, o que pode contribuir para os resultados ^26^. 

Apesar da elevada prevalência de percepção de saúde boa ou muito boa, indivíduos com obesidade apresentaram 47% mais chances de relatar percepção de saúde muito ruim, ruim ou regular. Em 2008, indivíduos com obesidade apresentaram chances 26% maiores (IC95% 1,07; 1,49) de apresentar percepção de saúde razoável ou ruim, comparando-os àqueles em eutrofia ^18^. Isso evidencia prejuízos que a obesidade produz na vida dos indivíduos ^4^. 

A obesidade relaciona-se à maior prevalência de doenças crônicas não transmissíveis, ao declínio da qualidade de vida, à redução do funcionamento físico, à limitação física, à dor e à redução da vitalidade, afetando a capacidade do indivíduo de viver plenamente a vida ^4^. Também aumenta a insatisfação com a imagem corporal, isolamento social, discriminação, depressão e ansiedade ^4^. 

O estigma vivenciado pelo indivíduo com obesidade se associa à inatividade física, ao comportamento alimentar nocivo, ao aumento de peso e às dificuldades para acessar serviços de saúde, potencializando os prejuízos à saúde ^4^. Tais efeitos podem ser mitigados em longo prazo com redução do peso. Na Suécia, de 2002 a 2014, entre 2.816 indivíduos de 30 a 75 anos, o maior índice de massa corporal no início de uma pesquisa predisse a percepção de saúde muito ruim, ruim ou regular dez anos depois entre quem manteve ou ganhou peso ^16^. 

Apesar da expansão do Programa Academia da Saúde, parte das ações de manejo da obesidade realizadas na atenção primária à saúde restringe-se às unidades básicas de saúde. Entretanto, verifica-se baixa adequação dessas unidades para o manejo adequado da obesidade, apresentando limitações quanto a estrutura física, equipe ampliada, registro dos usuários, entre outros ^27^. A qualificação profissional é outro limitante, apontando-se escassez de matriciamento, dificuldades para realizar diagnóstico e abordagem coletiva aplicada ao manejo da obesidade como importantes obstáculos para o manejo da obesidade ^28^. 

A prevalência de obesidade graus II ou III foi de aproximadamente 6% e 2%, respectivamente. Pessoas com obesidade graus II e III relataram dificuldade de acesso ao tratamento no SUS, incluindo baixa disponibilidade de recursos e preparo insuficiente das equipes para a abordagem da obesidade ^29^. O Programa Academia da Saúde pode não alcançar esse público, considerando que parte do ingresso ao serviço ocorre pelo referenciamento do usuário partindo da atenção primária à saúde ^14^.

O acompanhamento integral dos indivíduos com obesidade no SUS é premente, considerando a presença de outras doenças crônicas não transmissíveis, agravamento da obesidade progressivamente e suas associações com desfechos negativos em saúde ^4^. Considerando a sensibilidade da percepção de saúde para questões subjetivas, adotá-la em avaliações longitudinais de indivíduos com obesidade na atenção primária à saúde pode contribuir para formular e avaliar ações de promoção, prevenção e recuperação da saúde visando cuidado integral e qualidade de vida da população com obesidade ^5^. Aponta-se a importância da expansão e qualificação do Programa Academia da Saúde no país e de maior integração com as redes de atenção à saúde visando reduzir desigualdades de acesso e promover a melhoria da saúde da população, incluindo indivíduos com obesidade ^11^.

Destacaram-se como pontos fortes deste estudo o tamanho amostral, o rigor metodológico e controle da qualidade dos dados, e a validade interna dos resultados. 

A partir destes resultados, concluiu-se que os participantes do Programa Academia da Saúde apresentaram elevada prevalência de percepção de saúde boa ou muito boa, entretanto a presença de obesidade esteve associada a maiores chances de percepção de saúde muito ruim, ruim ou regular.

## Data Availability

O conteúdo do banco de dados está disponível mediante solicitação ao autor correspondente.
